# Effect of a combined household-level piped water and sanitation intervention on reported menstrual hygiene practices and symptoms of urogenital infections in rural Odisha, India

**DOI:** 10.1016/j.ijheh.2021.113866

**Published:** 2022-01

**Authors:** Belen Torondel, Jane Ferma, Suzanna C. Francis, Bethany A. Caruso, Parimita Routray, Heather Reese, Thomas Clasen

**Affiliations:** a London School of Hygiene and Tropical Medicine, Kepple Street, WC1E 7HT, London, UK; b Gangarosa Department of Environmental Health, Rollins School of Public Health, Emory University, 201 Dowman Dr, Atlanta, GA, 30322 Atlanta, GA, USA; c Hubert Department of Global Health, Rollins School of Public Health, Emory University, 201 Dowman Dr, Atlanta, GA, 30322, Atlanta, GA, USA

**Keywords:** Menstruation, WASH intervention, Urogenital symptoms, Odisha, India

## Abstract

Adequate menstrual hygiene management (MHM) requires access to water and sanitation and can be challenging for many women and girls living in resource-poor settings. Inadequate MHM has been associated with urogenital infections. The aim of this study is to assess the impact of a combined household-level piped water and sanitation intervention on MHM practices and urogenital infection symptoms (UGS) among women living in rural communities of Odisha (India). This study was nested within a pair-matched cohort study designed to assess impact of the Gram Vikas MANTRA program, which provided household-level piped water, bathing areas and latrine to all households in intervention villages, on diarrheal disease (primary outcome). The program did not specifically promote menstrual hygiene practices. Forty-five intervention villages were randomly selected from a list of those where implementation was previously completed at least five years before and matched to 45 control villages. Data for the main study was collected in four rounds from June 2015 to October 2016. For the MHM sub study, household surveys were administered in round four to randomly selected women aged 18 or older among study households from the 90 villages, to assess self-reported MHM practicesand urogenital infections symptoms. MHM practices were deemed adequate if they met some of the criteria developed on the basis of international monitoring that the GV program could modify (adequate frequency of absorbent change, washing the body with soap and privacy for managing menstruation). Multilevel mixed-effects logistic regression with a random effect distribution at the level of the pair and village was used to estimate the effect of the intervention on adequate MHM practices (primary outcome) and reported UGS (secondary outcome). A total of 1045 women (517 from intervention and 528 from control) were included in the study. Women who lived in the villages receiving the intervention, were more likely to report adequate MHM practices than those in control villages (Adjusted OR (AOR) 3.54, 95% Confidence Interval (CI): 1.86–6.78). 14.51% and 15.53% of women living in the control and intervention villages reported having at least one UGS. There was no evidence of an intervention effect on reported UGS (AOR = 0.97, 95%CI: 0.64–1.46). While household latrines or bathing areas with access to piped water improve the environment that enable MHM practices related to privacy, the provision of such facilities alone had only a moderate impact in adequate MHM and did not have an effect on self-reported UGS. More targeted inventions that include behavior change strategies and that address other barriers may be necessary to improve MHM practices.

## 1 Introduction

Every day, more than 300 million girls and women between the ages of 15 and 49 are menstruating(George, 2013). Menstrual hygiene practices are influenced by factors at the individual, family, and community level. The ability of girls and women to adequately manage their menstruation hygienically and with dignity is crucial to their health and well-being, and is a public health issue(Plesons et al., 2021; Sommer et al., 2015). A working definition of adequate menstrual hygiene management (MHM) was established by the WHO/UNICEF Joint Monitoring Programme on Water and Sanitation (JMP) in 2012 to inform global monitoring. MHM was defined as ‘Women and adolescent girls using a clean menstrual management material to absorb or collect blood that can be changed in privacy as often as necessary for the duration of the menstruation period, using soap and water for washing the body as required, and having access to facilities to dispose of used menstrual management materials’(Sommer and Sahin, 2013). The definition includes different aspects of the physical requirements for having adequate MHM, and it is increasingly being used among researchers and practitioners; however, a unified or standardized definition has not been achieved yet. Based on the WHO definition, Hennegan and colleagues developed a tool to quantify the different aspects of MHM and creating a single estimated that measure adequate MHM. Using this tool, they estimated the prevalence of adequate MHM among Ugandan school girls and found 90.5% of girls failed to meet available criteria for adequate MHM(Hennegan et al., 2016). Qualitative research in rural Odisha, India, which examined women's detailed accounts of menstruation at various life stages, proposed a revised definition of adequate MHM that captured voiced needs more comprehensively for women in the population(MacRae, Clasen, Dasmohapatra and Caruso, 2019b). Most of the components of the MHM definition—both the JMP's and revised versions—require appropriate resources and access to water and sanitation facilities to promote and facilitate adequate hygienic and comfortable menstrual practices. However, access to household sanitation and water remains a global challenge. As of 2015, 2.3 billion people lacked even basic sanitation services, with 860 million using unimproved facilities and another 890 million practicing open defecation—with a high proportion residing in India(World Health Organization, 2019). Due to this situation, government of India has made big investments in different sanitation campaigns during the last decade (World Bank, 2010) being the main focus toilet construction, with fewer resources availed for sustained coverage and use and without much attention to women needs (Garn et al., 2017). Despite finding good coverage levels of improved community water sources around rural India, the amount of water provided may be insufficient to fulfill the different needs that women face when managing their menstruation (Sebastian et al., 2013). Making water available into households, especially closer to the sanitation facilities may help to meet the needs that women require during menstruation (Routray et al., 2015; Schmidt et al., 2009). Several studies conducted in rural India have also emphasized that the lack of adequate sanitation at home influences women's experiences of safety and privacy(Caruso et al., 2015; Hulland et al., 2015; Sahoo et al., 2015; Sclar et al., 2018), and may impact mental health(Caruso et al., 2017, 2018).

Although water and sanitation are important for all women, the need for water, sanitation and hygiene (WASH) facilities in India is particularly urgent for those menstruating, including for personal washing and changing, and to meet the needs of the large number of women who use reusable materials that require washing. In India, between 43% and 88% of women wash and reuse cotton cloths rather than use disposable pads(Dasgupta and Sarkar, 2008; Narayan et al., 2001). However, reusable material may not be well sanitized because cleaning is often done without soap and with unclean water. Above it, the social taboos and restrictions force drying indoors, or covered by other clothing, away from direct sunlight and open air(MacRae, Clasen, Dasmohapatra and Caruso, 2019a; Sahoo et al., 2015). Unhygienic washing, drying and storing practices are particularly common in rural areas of Odisha state and amongst women and girls in lower socio-economic groups(Caruso et al., 2017; Das et al., 2015; MacRae et al., 2019b; Torondel et al., 2018) and have been associated with urogenital infections (Das et al., 2015; Torondel et al., 2018).

Two hospital-based studies conducted by our group in Odisha in 2015 and 2018, showed that women diagnosed with vulvovaginal yeast infection were more likely to use reusable absorbent material and practice lower frequency of personal washing than those who were not diagnosed with vulvovaginal yeast infections. And among women reusing absorbent material, vulvovaginal yeast infections were more frequent in women who dried their menstrual material inside their house and who stored the cloth hidden in the toilet compartment(Torondel et al., 2018). Compared to women diagnosed with bacterial vaginosis (BV), women without BV were more likely to practice personal washing more frequently and change absorbent material in a toilet facility and report higher frequency of absorbent change (Das et al., 2015; Torondel et al., 2018).

Urogenital tract infections which comprise reproductive tract infections (RTI) and urinary tract infections (UTIs) are a major public health concern worldwide and are particularly common in low-income settings(Lanfranco and Alangaden, 2016; Wasserheit et al., 1989). RTIs can result in pelvic inflammatory diseases, infertility, adverse pregnancy outcomes, and increased susceptibility to HIV(Nagarkar and Mhaskar, 2015). UTIs are a significant cause of morbidity in females of all ages. Serious sequelae include frequent recurrences, pyelonephritis with sepsis, pre-term birth and complications caused by frequent antimicrobial use(Flores-Mireles et al., 2015). The prevalence of reported symptoms of RTI in different population-based studies in Indian women varied from (13%–55%) (Baker et al., 2017; Bhilwar et al., 2015; Krupp et al., 2007; Sciences, 2006). A study to determine the prevalence of community acquired-UTIs in rural Odisha, showed that prevalence in females was 45.2%(Dash et al., 2013).

Studies on the role of WASH in the context of MHM have focused primarily on girls and the school environment and access to menstrual hygiene products (van Eijk et al., 2016), and there is less information on, and attention to the needs of women and girls outside the school environment and the influence of having appropriate WASH into MHM practices (Hennegan et al., 2018; Sommer et al., 2016). Our previous studies in India assessing the relationship between WASH access, MHM and urogenital infections showed that places where women can manage menstruation-related washing in privacy and comfort are important for adequate MHM(Das et al., 2015; Torondel et al., 2018). This was affected by having access to WASH facilities at the household.

As mentioned earlier, Indian Government's efforts to improve shortfalls in rural water and sanitation have been focused on constructions of community water sources and toilets for selected households. However, deficiencies in water quality, quantity and coverage at the household and community levels, and low use of toilets inspired a novel approach to WASH delivery led by Gram Vikas, a local non-governmental organization in Odisha, India. Their approach provides household-level piped water connections contingent on full community-level toilet coverage(Reese et al., 2017). In other words, once all in the community have a toilet, Gram Vikas ‘turns on’ water that is piped to all households and toilets. Our group conducted an evaluation to assess the impact of the Gram Vikas program on diarrhea (primary outcome) and other health outcomes(Reese et al., 2019; Sinharoy et al., 2021). Although the program addressed two potential drivers for appropriate menstrual hygiene practices, such as access to water and privacy, it did not specifically focus on improving the menstrual health among women in the community. To date, no research has assessed the impact of a household-level WASH intervention on menstrual hygiene practices and the potential urogenital symptoms that could result from poor hygiene.

**Fig. 1:**
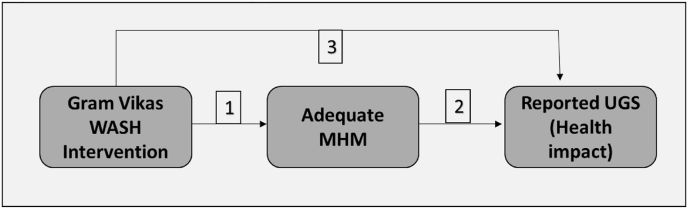
Theoretical model for the association between WASH, Adequate menstrual hygiene management (MHM) and reported urogenital infection symptoms (UGS). Objective 1 is to investigate the impact of WASH intervention on adequate MHM. Objective 2 is to investigate the association between adequate MHM and reported UGS. Objective 3 is to investigate the impact of WASH intervention on reported UGS.

We nested an MHM study within the evaluation of the Gram Vikas MANTRA program in rural Odisha, India. The objectives of this nested study were 1) Investigate the impact of WASH intervention on adequate MHM, 2) Investigate the relationship between adequate MHM and reported UGS and 3) Investigate the impact of WASH intervention on reported UGS. We also investigated the determinants of adequate MHM and reported urogenital symptoms (see Fig. 1).

## 2 Methods

### 2.1 The WASH intervention and evaluation

The MANTRA program (Movement and Action Network for the Transformation of Rural Areas), developed by Gram Vikas (Reese et al., 2017), consisted of: 1) a household pour-flush toilet with dual soak-away pits, 2) an attached bathing room, and 3) household piped water connections in the toilet, bathing room, and kitchen(Reese et al., 2017). For a village to be eligible to receive the program, every household must have committed to the construction of their own toilet and bathing room. Gram Vikas assisted with the development of a piped water system, which was connected after every household completed toilet construction, and the village assumed responsibility for the ongoing operation and maintenance costs.

The evaluation was of 45 matched villages with one intervention and one control village in each pair (90 total). The primary objective of the main study was to assess the long-term impact of the WASH intervention on diarrheal disease (Reese et al., 2019). The 45 intervention villages were randomly selected using a computer generated sequence from a list provided by Gram Vikas of villages with completed interventions in Ganjam and Gajapati districts, restricted to those with an intervention start date of 2003–2006. The intervention took an average of 3 years to be fully implemented; the latest year that a selected intervention village completed the implementation was in 2010(Reese et al., 2017). Between April–September of 2015, 45 control villages from the same districts, that had not received the Gram Vikas MANTRA WASH intervention, were matched retrospectively to the 45 intervention villages through a multi-step restriction, matching, and exclusion process to reduce potential bias due to baseline differences. We used an iterative multivariate matching scheme (R Matching package, version 4.9–2) to match villages on pre-intervention characteristics from the Government of India Census (2001) and Below Poverty Line Survey 2002, including demographic, socioeconomic, sanitation and water access characteristics, among others; balance was achieved on all variables(Reese et al., 2017). These village-level matching variables were selected due to their theorised association with the primary outcome, diarrhoeal diseases, as well as data availability. Villages were exact matched on district to limit any political or large-scale geographic variation between district populations (Diamond and Sekhon, 2013; Reese et al., 2017). Within each village, up to 40 households with children less than 5 years of age were randomly selected to be enrolled in the main evaluation study. The evaluation was carried out between June 2015 and October 2016 and consisted of four study rounds. Data collected in each round is described in Supplementary Fig. 1. Household and individual level data were collected during rounds 1 and 4 and included educational attainment of head of the household and primary caregiver, household wealth, health information of all the members of the house, and household access to sanitation and water (Supplementary Fig. 2).

### 2.2 MHM sub-study

The MHM sub-study was carried out during round four (July–Oct 2016). Sixteen households were randomly selected per village (1440 total), with one woman aged 18 years or older randomly selected to participate per household. Randomization of households and women was done using a computer generated sequence. Women were eligible to participate in the sub-study if they had experienced menstruation in the previous six months. The male and/or female household head provided written informed consent for the household and each participant consented before completing the MHM questionnaire. Individual level data for MHM practices and urogenital symptoms were collected in round four by female field workers in face-to-face interviews (Supplementary Fig. 3)**.** Data included marital status, age, menstrual practices (related to all the different domains captured in the MHM definition, including participant's body hygiene practices, type of absorbent used, and hygiene practices related to management of absorbent material), sanitation practices and urogenital symptoms. The questionnaire was adapted from a validated questionnaire that was used in a previous hospital based study conducted in Odisha to assess the association of different menstrual practices and urogenital infections(Torondel et al., 2018).

#### 2.2.1 Sample size

The sample size was based on objective 3, the effect of the WASH intervention on reported UGS. Our population-based study in Odisha showed that 13% of women reported at least one of the 4 urogenital symptoms used in our definition of UGS (Baker et al., 2017), and our hospital-based study in Odisha that investigated the effect of different household WASH characteristics on urogenital infections found that women whose water source was outside their home were 1.46 more likely to be a case of urogenital infection (defined with symptomatology) and 2.1 more likely to be laboratory diagnosed with BV or UTI infections compared with women who had the water source inside their home (95%CI 1.0–2.2) and (95%CI 1.3–3.4), respectively(Torondel et al., 2018). Therefore, we assumed that the WASH intervention could have an effect size of 0.55 on participants’ reported symptoms of UGS. For objective 3, if 13% had a reported urogenital symptom in the control group, with an effect size of 0.55, 80% power and 0.05 significance level, then at least 448 would be needed in each arm. We targeted 16 women per village (total 1440) to account for non-responses (due to not fitting into the eligibility criteria or refuse to participate) or absence the day of the visit.

### 2.3 Data management and analysis

Survey data was collected on mobile phones using the Open Data Kit (available from https://opendatakit.org/).

#### 2.3.1 Definitions of the outcomes and other covariates

The primary outcome for this study was ‘adequate MHM’ which was modified *a priori* from the Hennegan tool to quantify adequate MHM based on the working definition of MHM developed by WHO and UNICEF Joint Monitoring Programme (Sommer and Sahin, 2013) (Hennegan et al., 2016). The tool includes the domains of access to clean absorbents including (when relevant) sufficient washing, drying, storage and wrapping of reusable absorbents; adequate frequency of absorbent change; washing the body with soap and water; adequate disposal, and privacy for managing menstruation. However, the Gram Vikas WASH intervention could only affect certain domains of the definition, because it did not provide menstrual hygiene materials such as disposable pads or menstrual cups or methods for absorbent disposal. Therefore, we modified the tool to include only the domains from the MHM definition that the intervention could impact, such as adequate frequency of absorbent change; washing the body with soap and privacy for managing menstruation (Supplementary Table 1).

The secondary outcome was reported urogenital symptoms. After conducting a literature search of the most common symptoms used to diagnose RTIs (Balamurugan and Bendigeri, 2012; García et al., 2004; Prabha et al., 2012) and UTIs(Schmiemann et al., 2010), we selected four symptoms to assess reported urogenital symptoms: abnormal vaginal discharge; burning or itching in the genitalia; urinate more frequently; and burning or itching while urinating. Women were asked about current symptoms at the time of interview. If a woman reported at least one of the symptoms, then she was defined as having reported UGS positive(Das et al., 2015).

Potential confounding covariates were identified *a priori* based on the literature. Other variables we included were limited to what was collected in the overall evaluation of the WASH intervention. They included wealth index, healthcare decisions (who usually makes decisions about healthcare for yourself: you, someone else, or you and someone else decide jointly?), market access (How often do you go to the market/haat/bazaar?) and experience of stigma (Worried about being treated as untouchable by others). A wealth index variable was created(Reese et al., 2019). We used principal components analysis (R psych package, version 1.6.12) to construct the household wealth index from 15 variables, including household asset ownership, housing characteristics, agricultural land acreage, and below poverty line status(Bassani et al., 2014; Filmer and Pritchett, 2001). We extracted the component which explained the most variability as the wealth index(Kolenikov and Angeles, 2004).

#### 2.3.2 Statistical analysis

All analysis was conducted in STATA 15.1. Participant characteristics, MHM practices, and reported UGS were analyzed to provide descriptive statistics of the analytic sample.

To investigate the determinants of adequate MHM and reported UGS, two multilevel mixed-effects logistic regression models were carried out using methods for a matched-pair cluster randomised controlled trial(Hayes and Moulton, 2017). Unadjusted results were calculated for selected characteristics. The adjusted analysis included age *a priori* with other variables that were associated with the outcome using a p-value cut-off of 0.05 in the unadjusted analysis. Also, the effect of clustering and multicollinearity was assessed by evaluating the standard errors in each of the models.

To assess the impact of the WASH intervention on adequate MHM (Objective 1) we used the same multilevel mixed-effects logistic regression methods described above(Hayes and Moulton, 2017). While villages were exact matched by district and pre-intervention demographic, socioeconomic, sanitation and water access characteristics(Reese et al., 2017), there was likely to be confounding at the individual level. A crude estimate was calculated. Then an adjusted model was developed using age as a forced variable and adding each potential confounder identified in the determinant's analysis described above one by one (confounder + exposure + outcome). Effect estimates were compared to the crude estimates, and potential confounders were included in the final adjusted model if there was a 10% change from the crude estimate. The effect of clustering and multicollinearity was assessed by evaluating the standard errors in each of the models.

A similar analysis was used to assess the relationship between adequate MHM and reported UGS (Objective 2), and the effect of the WASH intervention with reported UGS (Objective 3).

Missing data were explored to investigate patterns and difference between the intervention groups using Chi square (Supplementary Table 2). In all the multivariate models, data was missing by design which resulted in excluded data in the analysis. Specifically, data was missing because some data was collected in the household portion of the survey, yet not all women responded to the HH portion of the survey because it targeted the mother or primary caregiver of the youngest child <5. Therefore, the women who responded to the MHM survey may not have responded to the HH survey. Thus data was missing at random (MAR).

## Ethical approval

The study was approved by LSHTM, U.K (No. 9071) and the Kalinga Institute of Medical Sciences of KIIT University, Bhubaneswar, India ethics committees (KIMS/KIIT/IEC/053/2015). The study was registered at ClinicalTrials.gov (NCT02441699).

## 3 Results

### 3.1 Characteristics of the study population

A total of 1440 women were visited in the 90 villages, and 395 were excluded from the study: 240 (16.6%) had reached menopause, 52 (3.6%) were pregnant, and 102 (7.0%) just gave birth. A total of 1045 women were included in this study, 528 were from control villages and 517 were from intervention villages. Table 1 presents participant socio-demographic characteristics. The mean age of the sample was 27 years (SD: 6.1) with most women aged 25–29 in both the control villages (35.2%) and intervention villages (38.3%). A higher proportion of women living in both the control (96.8%) and intervention (97.9%) villages were married. Ninety-eight percent of the sample were Hindu, with the remaining 2% Christian. Women living in the intervention villages were similar to women living in the control villages in regard to most sociodemographic variables. However, women in the intervention arm were wealthier than those in the control arm (24.4% vs 15.7%, respectively) and more women in the intervention arm had completed secondary school than those in the control arm (59.8% vs 44.3%, respectively). Women in the control arm were more likely to be caregivers who completed primary school or below than those living in the intervention arm (40.3% vs 30.0%, respectively). Women in the control arm reported having experienced stigma when menstruating more often than those in the intervention (36.7% vs 30.2%, respectively).

**Table 1:** Socio-demographic characteristics of women participating in the MHM study (N = 1045).

Characteristics		Women living in control villages *(n = 528)*		Women living in intervention villages *(n = 517)*	
		n^a^	% a	n^a^	%^a^
Age (years) *(n = 957)*	18–24	144	27.27	144	27.85
	25–29	187	35.42	198	38.30
	30 +	148	28.03	136	26.31
Religion *(n = 915)*	Christian/other	7	1.33	8	1.55
	Hindu	449	85.04	451	87.23
Marital Status *(n = 1045)*	Single	14	2.65	7	1.35
	Married	511	96.78	506	97.87
	Widowed	3	0.57	4	0.77
Wealth Index *(n = 832)*	Poor/Middle	323	61.17	300	58.03
	Rich	83	15.72	126	24.37
Caregiver Education Attainment *(n = 911)*	Primary or less	213	40.34	155	29.98
	Secondary or above	234	44.32	309	59.77
Experience Stigma^b^*(n = 1039)*	No	329	62.31	360	69.63
	Yes	194	36.74	156	30.17
Market Access c*(n = 908)*	No	109	20.64	121	23.40
	Yes	343	64.96	335	64.80
Healthcare Decision d*(n = 909)*	Self	133	25.19	124	23.98
	Someone else	152	28.79	154	29.79
	Self and someone else (joint)	167	31.63	179	34.62

a Numbers/percentages do not add up to 100% due to missing values. For further information on missing values see Supplementary Table 2.

b Menstruating women who experienced stigma from others during the last two menstruation cycles.

c The number of times menstruating women have attended the market. This indicates access to resources such as absorbents.

d Independence on healthcare decision making. This indicates ease of access to healthcare for women.

Missing data were identified and presented in Supplementary Table 2. The exposure of interest (WASH) and outcome of interest (UGS and MHM) had no missing data, however, MHM criterion variables consisted of some missing observations. Variables with more than 10% missing data were wealth index (20.4%), female caregiver education attainment (12.8%), healthcare decisions (13.0%), market access (13.1%) and religion (12.4%). Women living in the control villages vs. women living in the intervention villages were missing more data on wealth index (23.1% vs 17.6% p < 0.001), female caregiver attainment (15.3% vs 10.3% p < 0.0001) and experience of taboo (1% vs 0.2% p = 0.02).

### 3.2 A description of MHM practices

Table 2 shows the prevalence of the different menstrual hygiene practices, distributed in the five domains used by WHO to define MHM, among women from control and intervention arms.

**Table 2:** Self-reported MHM practices by study arm among women in Odisha, India during July–October 2016 (N = 1045).

Survey question	Survey responses	Women living in control villages *(n = 528)*		Women living in intervention villages *(n = 517)*	
		n^a^	%^12^	n a	% a
MHM Criteria 1: Clean absorbents					
What was the most commonly absorbent material used during the last 6 cycles? *(n = 1039)*	Disposable sanitary pads	48	9.09	92	17.79
	Reusable cloths/towel	473	89.58	422	81.62
	Nothing	1	0.19	1	0.19
	Other	1	0.19	1	0.19
With what do you wash your cloth? (n = 870)*	Water only	8	1.52	7	1.35
	Water and soap	449	85.04	404	78.14
	Other	1	0.19	1	0.19
After washing it, how do you dry the cloth? (n = 870)*	Dry it in the sun or open space	431	81.63	384	74.27
	Dry it inside the house	26	4.92	28	5.42
	Other	1	0.19	0	0.00
Where do you normally store the cloth for use next time? (n = 870)*	With my clothes	2	0.38	2	0.39
	In some place in the toilet	16	3.03	61	11.80
	Changing room or hidden place inside house	251	47.54	184	35.59
	In hidden place outside house	184	34.85	161	31.14
	Other	5	0.95	4	0.77
Do you wrap cloth in anything when storing? (n = 870)*	Yes, polythene	443	83.90	404	78.14
	Yes, other material	11	2.08	3	0.58
	No	4	0.76	5	0.97

MHM Criteria 2: Adequate frequency of absorbent change					
How often do you change your absorbent material on your heaviest day? *(n = 1039)*	1x per day	21	3.98	30	5.80
	2x per day	247	46.78	252	48.74
	3x per day	165	31.25	151	29.21
	4x per day	59	11.17	50	9.67
	5+ times per day	31	5.87	33	6.38

MHM Criteria 3: Washing the body practices					
What type of washing do you practice during menstruation? *(n = 1039)*	Only vaginal wash	178	33.71	165	31.91
	Bath of full body	341	64.58	349	67.50
	I don't wash myself	4	0.76	2	0.39
How often do you wash yourself (bath or vaginal wash) during menstruation? *(n = 1032)*	Only the first day of my cycle	14	2.65	21	4.06
	A few times throughout the cycle	79	14.96	74	14.31
	At least once every day	20	3.79	21	4.06
	More than once everyday	405	76.70	398	76.98
What do you use to wash yourself during menstruation? *(n = 1031)*	Water only	16	3.03	17	3.29
	Water and soap/detergent	501	94.89	496	98.94
	Other	1	0.19	0	0.00

MHM Criteria 4: Difficulty with disposal					
Had difficulty finding a place to dispose of cloth or pad during your last two menstrual periods? *(n = 1039)*	Never	487	92.23	485	93.81
	Sometimes	17	3.22	19	3.68
	Always	19	3.60	12	2.32

MHM Criteria 5: Privacy for managing menstruation					
Where do you most often change your absorbent material when at home? *(n = 1039)*	In household toilet	26	4.92	141	27.27
	Bathing room	22	4.17	100	19.34
	In toilet of neighbour/relative	0	0.00	1	0.19
	In private room in the house	405	76.70	253	48.94
	Outside (field/rive/pond etc)	59	11.17	20	3.87
	Other	11	2.08	1	0.19
Where do you wash the absorbent materials you reuse? (n = 870)*	Inside toilet stall	24	4.55	222	42.94
	Bathroom	14	2.65	38	7.35
	At private tube well/tap in yard or house	17	3.22	5	0.97
	At public tube well/tap in village	8	1.52	0	0.00
	In pond/river	353	66.86	116	22.44
	I do not wash it/NA	1	0.19	0	0.00
	Other	41	7.77	31	6.00

a Numbers/percentages do not add up to 100% due to missing values for further information on missing values see Supplementary Table 2 *Answered only by women who said that reusable cloth was the most common material used in the last 6 months.

#### 3.2.1 Clean absorbents

The type of menstrual absorbent most commonly used during the last six cycles in this community was a reusable cloth/towel. More women living in the control arm used reusable materials than women living in the intervention arm (89.6% vs 81.6%), and disposable sanitary pads were more commonly used in intervention arm than in control arm. Among women reusing cloths/towels, more women in the control arm washed them with soap and water and dried them in the sun or in an open space compared with women from intervention arm (85% vs 78.1% and 81.6% vs 74.3%, respectively). More women in the control arm stored their cloth in a changing room or hidden inside the house or hidden at a place outside the house than the intervention arm (47.5% vs 35.6% and 34.9% vs 31.1%, respectively). The proportion of women reporting storing their reusable cloth/towel with other clothes was very low in both arms (0.4% and 0.4%, respectively). More women living in the control arms wrapped their cloth in polythene when they stored their reusable cloth than women living in the intervention arms (83.9% vs 78.1%).

#### 3.2.2 Adequate frequency of absorbent change

Adequate frequency of absorbent change is defined as women who change their absorbent three times or more per day on their heaviest day(Das et al., 2015). There was no evidence of a difference in frequency of absorbent change between the women living in the intervention and control arms (45.2% and 48.2%, respectively).

#### 3.2.3 Washing the body practices

Three quarters of women reported that they practice full body washing during menstruation and a third reported only doing a vaginal wash. Most women reported washing with water and soap during their menstruation with no evidence of a difference of these practices between women living in the control and intervention arms (94.9% vs 98.9%). Most women reported washing more than once every day during their menstruation and no difference was found between women living in the control and intervention arms (76.7% and 76.9%, respectively).

#### 3.2.4 Difficulty with disposal

Most women reported that they did not have difficulty in finding a place to dispose the cloth or pad during the last two menstrual periods. There was no evidence of a difference between women living in control and intervention arms (92.2% vs 93.8%).

#### 3.2.5 Privacy for managing menstruation

The location where women changed their absorbents differed between control and intervention arm. A higher proportion of women in the control arm reported changing their absorbent material in a private room in the house compared to women living in the intervention villages (76.7% vs 48.9%). Fewer women in the control villages changed their absorbent material in the household toilet or bathing room compared to women in the intervention arm (4.9% vs 27.3% and 4.2% vs 19.3%, respectively). More women living in the control arm changed their absorbent outside (hiding behind a bush/tree in the open field/river/pond etc) than women living in the intervention arm (11.2% vs 3.9%).

More women in the intervention arm washed their absorbent inside the toilet stall or bathroom than women living in control arm (42.9% vs 4.6% and 7.4% vs 2.7%, respectively). More women living in the control arm wash their absorbents in a pond/river than women living in the intervention arm (66.9% vs 22.4%, respectively).

### 3.3 Prevalence and determinants of adequate MHM

Using the modified definition of adequate MHM, 10.1% of participants had adequate MHM: 4.7% among women living in control arm; and 15.7% living in the intervention arm. Supplementary Table 3 displays the results of the unadjusted and adjusted analyses of selected characteristics and the adequate MHM definition. The adjusted analysis showed that wealth index and female caregiver education attainment were independently associated with adequate MHM. Women who were wealthier had 1.88 times the odds of adequate MHM compared to women who were poorer (95% CI:1.08–3.26). Women whose caregiver completed education at the secondary school level and above, had 2.33 times the odds of adequate MHM compared to women whose caregiver completed education at primary level and below (95% CI:1.20–4.53). There was a weak association with the experience of stigma and MHM; women who had experienced stigma had 1.65 times the odds of adequate MHM than women who did not experience stigma (95% CI:0.96–2.85). No other variables were independently associated with the relaxed definition of MHM.

### 3.4 Prevalence and determinants of reported UGS

Supplementary Table 4 describes the prevalence of reported urogenital symptoms in the MHM sub-study. Abnormal vaginal discharge was the most reported symptom (9%). Women in the intervention villages had less burning or itching in the genitalia two weeks prior to the survey than women in the control villages (2.9% vs 5.7%, p = 0.03). There was no evidence of other differences in reported UGS between women living in control and intervention villages.

Based on the combined reported UGS variable (having at least one symptom), 15.0% women reported symptoms of diseases pertaining to a urogenital infection. Supplementary Table 5 displays the results of the unadjusted and adjusted analyses of selected characteristics and the UGS outcome. Based on univariate and multivariate analysis none of the characteristics showed strong evidence of an association with the combined UGS variable. There was no evidence of multicollinearity in the multivariable models.

### 3.5 The effect of the WASH intervention on adequate MHM

Table 3 displays the effect of the WASH intervention on adequate MHM. Women living in intervention villages had 3.82 times the odds of adequately managing their menstruation compared to women living in the control villages (95% CI: 2.25–6.50) in the unadjusted analysis. After adjusting for age, caregiver education attainment, experience of stigma and wealth index, women living in the intervention villages had 3.54 times the adjusted odds of adequately managing their menstruation compared to women living in the control villages (95% CI: 1.86–6.78).

**Table 3:** The effect of the WASH intervention on adequate MHM among menstruating women living in Odisha, India July–October 2016 (N = 1045).

		**Adequate MHM**^a^				
			**Unadjusted**^b^*(n = 1045)*		**Adjusted**^c^*(n = 743)*	
	**n event/N (%)**		**Odds Ratio (95% CI)**	**P-value**^d^	**Odds Ratio (95%CI)**	**P-value**^d^
Women living in control villages		25/528 (4.7)	1 –	<0.001	1 –	<0.001
Women living in intervention villages		81/517 (15.7)	3.82 (2.25–6.50)		3.54 (1.86–6.78)	

a Adequate menstrual hygiene practices definition: Adequate frequency of absorbent change, Wash body with soap and water (frequency and type of washing only) and privacy for managing menstruation.

b Adjusted for clustering at the pair and village level.

c The model was adjusted for clustering at the pair and village level, age and for variables that changed the OR by >10% in bivariate models including wealth index, female caregiver education attainment and experience of stigma (Supplementary Table 1). Sample size decreased due to missing data in the confounder variables. Data was missing because not all women responded to the HH survey as the HH survey targeted the mother or primary caregiver of the youngest child <5. Missing data were explored to investigate patterns and difference between the intervention groups using Chi square (Supplementary Table 2).

d P-values derived from nested likelihood ratio tests.

### 3.6 The effect of the WASH intervention on reported UGS

14.51% and 15.53% of women living in the control and intervention villages reported having at least one symptom of UGS. Women living in intervention villages had 0.92 times the odds of a reported UGS compared to women living in the control villages (95% CI: 0.66–1.37) in the unadjusted analysis (Table 4). After adjusting for experience of stigma and caregiver education attainment, women in the intervention villages had 0.97 times the odds of self-reporting symptoms (95% CI: 0.64–1.46; p = 0.9) than women living in the intervention villages. Having adequate MHM practice was not associated with UGS symptoms (0.81; 95%CI: 0.39–1.68 p = 0.6) when adjusted by age (Table 4).

**Table 4:** The effect of the WASH intervention on reported urogenital symptoms (UGS) and effect of adequate MHM on UGS among menstruating women living in Odisha, India July–October 2016 (N = 1045).

		The combined UGS variable a						
				**Univariate**^b^*(n = 1045)*		**Multivariate**^c^		
			**n event/N (%)**	**Odds Ratio (95% CI)** **P-value**^d^		**N**^e^	**Odds Ratio (95%CI)**	**P-value**^d^
WASH	Women living in control villages		82/528 (15.5)	1 –	0.8	901	1 –	0.9
	Women living in Intervention villages		75/517 (14.5)	0.92 (0.66–1.37)			0.97 (0.64–1.46)	
MHM f	Women who have inadequate MHM		143/939 (15.2)	1 –	0.6	957	1 –	0.6
	Women who have adequate MHM		14/106 (13.2)	0.84 (0.45–1.55)			0.81 (0.39–1.68)	

a The combined UGS variable consists of self-reported symptoms in the past two weeks of abnormal vaginal discharge, burning or itching in the genitalia, burning or itching when urinating and urinating frequently.

b Adjusted for clustering at the pair and village level.

c The model was adjusted for clustering at the pair and village level, age and variables that changed the OR by >10% in bivariate models. For the WASH variable this includes: Experience of stigma and female caregiver education. For the MHM variable there were no identified confounders to adjust for (Appendix 5).

d P-values derived from nested likelihood ratio tests.

e Total number of women in the final model.

f Adequate MHM definition: Wash body with soap and water and privacy for managing menstruation.

## 4 Discussion

To the best of our knowledge, this is the first study to evaluate the effect of an intervention designed to improve water and sanitation at the household level on MHM practices and urogenital symptoms. The study findings support the hypothesis that the Gram Vikas WASH MANTRA intervention is associated with better MHM practices. Women living in the intervention villages reported more adequate MHM practices related to privacy aspects of changing and washing compared to women living in control villages. More women living in the intervention villages changed the absorbent in the household toilet and bathing room; whereas more women living in the control villages changed the absorbent outside (e.g. field, river, pond). Among women who reused the cloth, women in control villages washed their material in the pond or river three times more than women in intervention villages, whilst women in intervention villages washed their menstrual absorbents five times more often inside the toilet stall or bathing room compared to women in control villages. All these differences can be explained by the novelty of this intervention in providing toilets and bathrooms with piped water, which is not typical of WASH interventions in India or elsewhere.

Interestingly, a fifth of women in intervention villages still use ponds to wash their material, despite having a toilet or bathroom constructed at home. A similar result was observed in a qualitative study conducted in another rural district from the same state, which showed that only 20% of the interviewed women washed their absorbent in the latrines(MacRae et al., 2019b). The persistence of these washing practices could be explained by the socializing habits that women from rural communities have when going to open defecation, especially in the evening, which is a rare opportunity for them to leave their houses and be free from household chores and responsibilities(Routray et al., 2015). Open defecation (OD) normally happens next to ponds and women probably change and wash their absorbent after practicing OD.

Similar practices were reported for adequate frequency of absorbent change and for washing the body with soap and water among both groups, suggesting that our intervention did not impact these aspects of MHM. We could hypothesize that having a private toilet and bathing area would increase the frequency of changes of absorbents; however, there was no difference in the frequency of absorbent change, indicating that this behavior could be influenced more by the type of material used, which the Gram Vikas MANTRA intervention did not attempt to change, and not much by the physical environment.

Therefore, privacy for managing menstruation appeared to be the biggest driver in the definition of adequate MHM. The findings of this study are consistent with previous studies that have shown that women who have access to WASH facilities have higher odds of adequately managing their menstruation than women who do not(Das et al., 2015; Hennegan et al., 2018). A qualitative study conducted in the same state reported that inadequate menstruation ranked as one of the most stressful sanitation behaviors for women’, forcing them to navigate social and physical barriers during their daily sanitation routines(Hulland et al., 2015). Still, follow-on research in Odisha found that women who lacked access to a functional latrine, an enclosed bathing space, or a water source within their compound, had significantly higher overall ‘Menstrual Insecurity’ scores—indicating greater insecurity—than those with these facilities(Caruso et al., 2020). These findings suggest that WASH infrastructure has inherent benefits to menstruators, even if fully adequate MHM may not be realized. As such, intervention that address components of the WASH environment alone, like the Gran Vikas MANTRA intervention may be impactful and necessary in changing menstrual practices, even if not sufficient to enable adequate MHM.

Even though there was evidence that the intervention improved MHM, the proportion with adequate MHM practices in the intervention arm was still low (16%), suggesting that other factors such as type of material used, or cultural and traditional habits related to changing and washing can influence some of the practices that infrastructure cannot change alone. In fact, another qualitative study conducted in the same state to understand women's menstruation-related concerns, indicated that in order to improve menstrual experiences more is needed than facilities that change the physical environment alone(Caruso et al., 2017). Efforts to enable urinating, defecating and managing menstruation independently, comfortably, safely, hygienically, privately, healthily, with dignity and as needed require transformative approaches that also address the gendered, sociocultural and social environments that impact women despite facility access.

There were several other independent predictors of adequate MHM. Women who were wealthier and who had educated caregivers were more likely to have adequate MHM, suggesting the importance of knowledge and resource access. Several studies conducted in India have also showed similar results(Kansal et al., 2016; Khanna et al., 2005), indicating that wealth and education of the mother are important predictors for following hygienic practices during menstruation. There was a weak association with the experience of stigma and increase adequate MHM. One potential explanation is that women that experience stigma could change their practices, which could lead to better hygienic practices.

The relationship between the WASH intervention, MHM and urogenital symptoms was less clear. Women living in the intervention villages reported less genital burning or itching compare to women living in control villages. This finding could be related to the type of menstrual material used, as women living in control villages used more reusable cloths. Previous studies conducted in Odisha, India found that women who use reusable pads were more likely to have urogenital symptoms than women who were using disposable sanitary pads(Das et al., 2015; Torondel et al., 2018). These previous studies also showed an association between changing in a toilet and reduced UGS. Our study did not find any association between reported urogenital symptoms between women living in the control or intervention villages, nor any association between women with adequate MHM and urogenital symptoms; however, we were limited by measuring reported symptoms only, genital symptoms can be poorly predictive of the urogenital infections(Anderson et al., 2004). More studies using laboratory diagnosed urogenital infections are needed to better understand health outcomes related to MHM and WASH interventions.

A growing number of studies in India have shown how access to sanitation may influence health beyond disease, particularly for women and girls(Caruso et al., 2018; Sclar et al., 2018). Different studies indicate that inadequate sanitation may put women and girls at greater risk of experiencing violence(World Health Organization, 2013). For example, an ethnographic study in urban slums in Pune and Jaipur documented the harassment and violence that women regularly face when going for open defecation(Kulkarni, O'Reilly and Bhat, 2017). Therefore, access to appropriate WASH when menstruating is very important for safety and mental health.

The strength of this study includes its design, a large matched-cohort that have received the WASH intervention since 2004. The match design provides rigorous means for estimating causal effects given that randomization to the intervention group was not feasible due to the several year implementation process(Arnold et al., 2010; Reese et al., 2017). While there are limitations inherent to observational studies, the matched study design and multivariable modelling analysis plan reduce the potential for confounding, and robust analytical methods were used to generate effect estimates(Reese et al., 2017). Another strength is that interviewers were women field workers and surveys were conducted in private spaces of the houses, which assured a relaxed environment to discuss a stigmatized topic.

There are several limitations. Firstly, the study outcomes of adequate MHM and symptoms were based on self-reported responses from the survey, which is subject to social desirability bias and recall bias. Secondly, the fact that we used a pool estimate to describe MHM, required to establish a predefined criterion to establish what was a good or bad practice for each component of the definition, which was based on very limited literature. The need for a pool estimate could be also questioned, as we believe it is useful to establish the state of MHM in different populations and use this data for advocacy, but other studies have argued that the definition did not capture other factors such as menstrual taboos or social support that can impact menstrual practices but are not captured in the definition(Hennegan et al., 2016; MacRae et al., 2019b). Hennegan also suggested that until evidence guidelines are developed, and comparable measures of MHM have been tested and used across studies, it is not advised to present only pooled estimates; it would be more informative to present the individual aspects that make up MHM to be able to understand relationships with outcomes(Hennegan et al., 2016). During the last years, the MHM definition has continued to evolve, and future studies should test other definitions to inspire how to assess adequacy(Hennegan et al., 2021; MacRae et al., 2019b). Thirdly, the study design presented certain limitations. As the main aim of the primary study aimed to asses longer-term effects, a study design in which the intervention status was not randomly assigned was employed (Reese et al., 2019). Despite both study arms being well balanced at the village-level after matching on pre-intervention characteristics, we still cannot rule out potential for residual confounding (Reese et al., 2019). In addition, we did not have available pre-intervention urogenital infections prevalence that could be used for the matching process. Another limitation is that we could not assess immediate impacts on the intervention of our outcomes of interest, due to the time lapse between intervention completion and study initiation (Reese et al., 2019). Finally, there are limitations to generalizability. Interventions study villages were randomly selected from those where the implementation was complete, however there were villages who refuse to participate when approached first time by Gram Vikas during their motivation visit. Despite these villages being excluded from the list of potential control villages, non-participating villages may be different from participating villages in their awareness of health risks, collective efficacy, or other characteristics, thus introducing selection bias(Reese et al., 2019). Therefore, this study cannot conclude that the Gram Vikas intervention can have the same impact observed in this study across all villages in this setting or elsewhere (Reese et al., 2019).

## 5 Conclusion

In conclusion, this study provides evidence that a combined intervention, where provision of household piped water connections were combined with community sanitation coverage, is important to improve environment that enable adequate MHM practices among women living in these communities. However, in order to achieve a higher impact on adequate MHM among women in these communities, more targeted interventions towards addressing other barriers to improve MHM are needed.

## Declaration of competing interest

This research does not contain any conflict of interests.

## Data statement

The datasets used and/or analyzed during the current study are available from the corresponding author on reasonable request.
