# Supplementary material for: Effect of a combined household-level piped water and sanitation intervention on reported menstrual hygiene practices and symptoms of urogenital infections in rural Odisha, India
Source: Int J Hyg Environ Health. 2022 Jan;239:113866. doi: 10.1016/j.ijheh.2021.113866 (PMC8669071; doi:10.1016/j.ijheh.2021.113866)
Supplement: Multimedia component 1 [file mmc1.docx]

# **Supplementary material**

**Supplementary Figure 1**: Matching criteria for selection of intervention and control villages and timeline for study rounds and outcomes data collection.


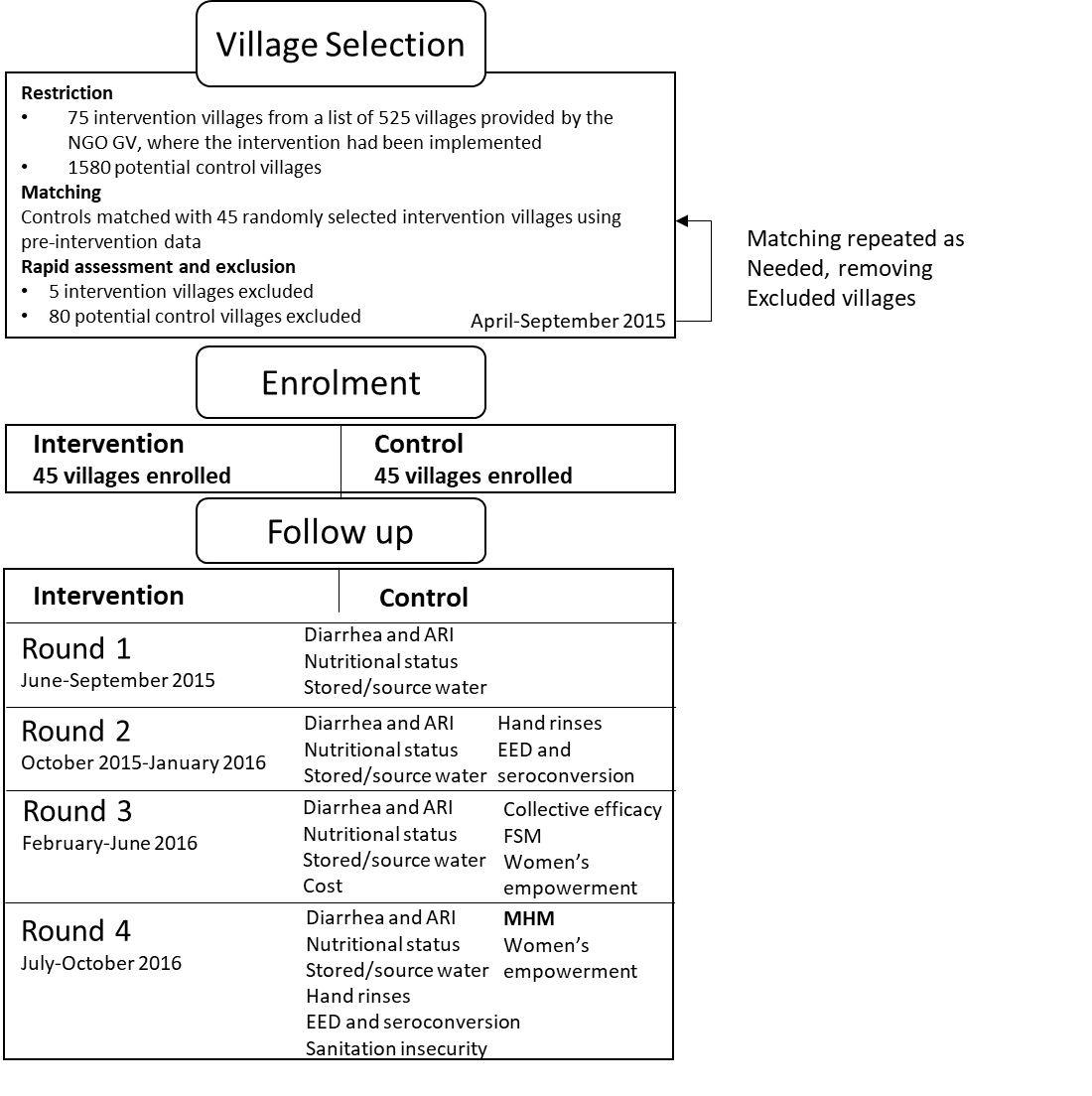


## **Supplementary Figure 2:** Household Survey

| **Household 4 Survey** | | | |
| --- | --- | --- | --- |
| **SECTION A: Eligibility & Demographics** | | | |
| Village ID: __  Hint: 1-90  **CODING NOTE:** Must be between 1 – 90. | | Household ID: __  Hint: 1-999  **CODING NOTE:** Must be between 1 - 999 | |
| Date of data collection: [DD/MM/YYYY] | | Survey start time: [hh:mm] | |
| **ENUMERATOR NOTE:** There is no R1/R2.R3 data for this household. Confirm that this is a new household. | | | **PROGRAMMING NOTE** |
| hh.rslt | Result | 1 = Completed  2 = Entire household absent or no competent respondent at home 3 = Survey completed, but child absent/unwilling for weight and height 4 = Drop out (unwilling to participate further in the study) |  |
| hh.respond | Select the respondent | [List of hh.m]  66 Other  77 NA |  |
| **SECTION B: Household Members**  Repeat for all current household members | | |  |
| hh.dob | What is [hh.name]’s date of birth? |  |  |
| wom.edu4 | [Female caregiver education, categorical] | 1 = <1 yr complete 2 = Completed primary (>=4 yrs) 3 = Completed secondary (10 yrs) 4 = More than secondary (>10 yrs) |  |
| hh.relig2 | [Religion, binary] | 0= Other/Christian 1= Hindu |  |
| hh.pov | [Combined GoI poverty card] | 1= Antodaya (HH income <Rs 250/mo) 2= BPL 3= Neither card |  |
| hh.ant | Do you have an Antodaya card? | 1 = Yes, verified 2 = Yes 3 = No 99 = Don't know |  |
| hh.bpl | Do you have a BPL card? | 1 = Yes, verified 2 = Yes 3 = No 99 = Don't know |  |
| **SECTION F: Household Activities**  Now I’d like to ask you some questions about your participation in certain types of activities. | | | Answer Section F if hh.respond is  NOT 77 |
| G3.01 | Who usually makes decisions about healthcare for yourself: you, someone else, or you and someone else decide jointly? | 1 Self  2 Someone else  3 Self and someone else decide jointly  98 NA |  |
| G6.02 | How often do you go to the market/ haat/ bazaar? | 1 Every day  2 Every week at least once  3 Every two weeks at least once  4 Every month at least once  5 Less than once a month  6 Never |  |
| **SECTION H: Household Water** | | |  |
| hh.rslt | Result  *Hint: Do not designate household as 'Drop out' until a supervisor returns to the household and confirms that they are dropouts* | 1 Completed  2 Entire household absent or no competent respondent at home  3 Survey completed, but child absent/unwilling for weight and height  4 Drop out (unwilling to participate further in the study)  5 Other |  |

## **Supplementary Figure 3:** MHM survey.

| **MHM/SS Survey** | | | |
| --- | --- | --- | --- |
| **SECTION A: Eligibility & Demographics** | | | |
| Village ID: __  Hint: 1-90  **CODING NOTE:** Must be between 1 – 90. | | Household ID: __  Hint: 1-999  **CODING NOTE:** Must be between 1 - 999 | |
| Date of data collection: [DD/MM/YYYY] | | Survey start time: [hh:mm] | |
| **ENUMERATOR NOTE:** There is no R1/R2.R3 data for this household. Confirm that this is a new household. | | | **PROGRAMMING NOTE** |
| **hh.respond** | Start from person ${rand.select}. Select the person who is responding. | [List of hh.m] |  |
| dem01 | What is your marital status? | 1 single  2 married  3 separated  4 divorced  5 widowed |  |
| **SECTION B: Concerns Regarding Sanitation** | | | Answer Section B if hmh.surv = 1 |
| lat04 | Where have you typically gone to urinate during the day in the past month? | 1 Household toilet  2 Neighbor's /relative's toilet  3 Bathroom  4 Field  5 Jungle  6 Roadside  7 By river/stream/pond  88 Other |  |
| **SECTION C: Menstruation and Child Birth**  Now we are going to discuss menstruation | | |  |
| mh01 | Do you currently experience menstruation/periods? | 1 Yes, regular  2 Yes, but irregular  3 No, menopause  4 No, pregnant  5 No, recently gave birth  6 No, have never experienced menstruation |  |
|  | How often have you experienced any of the following during your last two menstrual periods? |  | Answer if mh02a <=8 or mh02=1 |
| mi33 | Had difficulty finding a place to dispose of cloth or pad | 1 Never  2 Sometimes  3 Often  4 Always |  |
| mi42 | Worried about being treated as untouchable by others | 1 Never  2 Sometimes  3 Often  4 Always |  |
| mh03 | What was the most commonly absorbent material used during the last 6 cycles? | 1 Disposable sanitary pads  2 Reusable cloths/towel  3 Nothing  88 Other |  |
| mh05 | How often do you change your absorbent material on your heaviest day? | ___ |  |
| mh06 | Where do you most often change your absorbent material when at home? | 1 In household toilet  2 Bathroom  3 In toilet of neighbor/relative  4 In private room in the house  5 Outside (field/river/pond etc.)  88 Other |  |
| mh07 | Do you typically reuse your absorbent material after a single use or throw it away? | 1 Reuse  2 Throw it away |  |
| mh08 | What is the most common reusable material you use? | 1 Old cotton fabric (sari or other)  2 Old silk/nylon fabric (sari or other)  3 Towel  88 Other | Answer if mh02a <=8 or mh02=1, and mh07=1 and mh03=2 |
| mh09 | Where do you wash the absorbent materials you reuse?  *Hint: Select the main one* | 1 Inside toilet stall  2 Bathroom  3 At private tube well /tap in yard or in house  4 At public tube well /tap in village  5 In pond / river  6 I do not wash it/Not applicable  88 Other |  |
| mh10 | With what do you wash your cloth? | 1 Water only  2 Water and soap/detergent  88 Other |  |
| mh11 | After washing it, how do you dry the cloth?  *Hint: Select the main one* | 1 Dry it in the sun or open space  2 Dry it inside the house  3 I don’t dry it  88 Other |  |
| mh12 | Where do you normally store the cloth for use next time?  *Hint: Select the main one* | 1 With my clothes  2 In some place in the toilet  3 In some place of the changing room or hidden place inside house  4 In hidden place outside house  88 Other |  |
| mh13 | Do you wrap the cloth in anything when storing? | 1 Yes, polythene  2 Yes, other material  3 No |  |
| mh14 | Where do you dispose your sanitary pad (once you have finished using it)?  *Hint: Select the main one* | 1 Inside latrine  2 In a rubbish bin inside or close to the latrine  3 In the household rubbish bin  4 Put it in the pond /river/ stream  5 I burn it  6 Discard in any other open space  88 Other | Answer if mh02a <=8 or mh02=1, and mh07=2 |
| mh16 | What type of washing (bath or vaginal wash) do you practice during menstruation? | 1 Only vaginal wash  2 Bath of full body  3 I don’t wash myself | Answer if mh02a <=8 or mh02=1 |
| mh17 | How often do you wash yourself (bath or vaginal wash) during menstruation? | 1 I don’t wash myself  2 Only the first day of my cycle  3 A few times throughout the cycle  4 At least once every day  5 More than once everyday | Answer if mh02a <=8 or mh02=1, and mh16=1 or mh16=2 |
| mh18 | What do you use to wash yourself during menstruation? | 1 Water only  2 Water and soap/detergent  88 Other |  |
| sym01 | In the last two weeks, did you have abnormal vaginal discharge?  *Hint: Quantity, color, consistency or odor* | 1 Yes  2 No  99 Don’t know | Answer if mh01=1 or mh01=2 or mh01=5 |
| sym01a | Is this symptom recurrent (happening more than once)? | 1 Yes  2 No  99 Don’t know | Answer if mh01=1 or mh01=2 or mh01=5, and sym01=1 |
| sym01b | How often have these problems appeared in the last year approximately? | 4 Monthly  3 Three or four times per year  2 Twice per year  1 Once per year  99 I can not remember | Answer if mh01=1 or mh01=2 or mh01=5, and sym01a=1 |
| sym02 | In the last two weeks, do you have a feeling of burning or itching in the genitalia (vulvovaginal area)? | 1 Yes  2 No  99 Don’t know | Answer if mh01=1 or mh01=2 or mh01=5 |
| sym02a | Is this symptom recurrent (happening more than once)? | 1 Yes  2 No  99 Don’t know | Answer if mh01=1 or mh01=2 or mh01=5, and sym02=1 |
| sym02b | How often have these problems appeared in the last year approximately? | 4 Monthly  3 Three or four times per year  2 Twice per year  1 Once per year  99 I can not remember | Answer if mh01=1 or mh01=2 or mh01=5, and sym02a=1 |
| sym03 | In the last two weeks, do you present genital sores? | 1 Yes  2 No  99 Don’t know | Answer if mh01=1 or mh01=2 or mh01=5 |
| sym03a | Is this symptom recurrent (happening more than once)? | 1 Yes  2 No  99 Don’t know | Answer if mh01=1 or mh01=2 or mh01=5, and sym03=1 |
| sym03b | How often have these problems appeared in the last year approximately? | 4 Monthly  3 Three or four times per year  2 Twice per year  1 Once per year  99 I can not remember | Answer if mh01=1 or mh01=2 or mh01=5, and sym03a=1 |
| sym04 | In the last two weeks, do you feel the need to urinate more frequently? | 1 Yes  2 No  99 Don’t know | Answer if mh01=1 or mh01=2 or mh01=5 |
| sym04a | Is this symptom recurrent (happening more than once)? | 1 Yes  2 No  99 Don’t know | Answer if mh01=1 or mh01=2 or mh01=5, and sym04=1 |
| sym04b | How often have these problems appeared in the last year approximately? | 4 Monthly  3 Three or four times per year  2 Twice per year  1 Once per year  99 I can not remember | Answer if mh01=1 or mh01=2 or mh01=5, and sym04a=1 |
| sym05 | In the last two weeks, do you have a feeling of burning or itching or pain when urinating? | 1 Yes  2 No  99 Don’t know | Answer if mh01=1 or mh01=2 or mh01=5 |
| sym05a | Is this symptom recurrent (happening more than once)? | 1 Yes  2 No  99 Don’t know | Answer if mh01=1 or mh01=2 or mh01=5, and sym05=1 |
| sym05b | How often have these problems appeared in the last year approximately? | 4 Monthly  3 Three or four times per year  2 Twice per year  1 Once per year  99 I can not remember | Answer if mh01=1 or mh01=2 or mh01=5, and sym05a=1 |
| sym06 | In the last two weeks, do you have cloudy urine or blood in your urine? | 1 Yes  2 No  99 Don’t know | Answer if mh01=1 or mh01=2 or mh01=5 |
| sym06a | Is this symptom recurrent (happening more than once)? | 1 Yes  2 No  99 Don’t know | Answer if mh01=1 or mh01=2 or mh01=5, and sym06=1 |
| sym06b | How often have these problems appeared in the last year approximately? | 4 Monthly  3 Three or four times per year  2 Twice per year  1 Once per year  99 I can not remember | Answer if mh01=1 or mh01=2 or mh01=5, and sym06a=1 |
| sym07 | Did you change your menstrual hygiene habits after having any recurrent symptoms? | 1 Menstrual absorbent type  2 Vaginal washing practice  3 Body washing practices  4 Absorbent washing practices (if applied)  5 Place for changing menstrual absorbent  6 Place to defecate or urinate every day  7 Place to defecate or urinate during menstruation  8 No  Select all that apply | Answer if sym01a=1 or sym02a=1 or sym03a=1 or sym04a=1 or sym05a=1 or sym06a=1 |

**Supplementary Table 1.** Criteria and survey questions used for defining ‘Adequate menstrual hygiene management (MHM)’.

| MHM Criteria | Requirements | Survey Question | Survey responses | Adequate MHM definition  (0 = inadequate MHM  1 = Adequate MHM) |
| --- | --- | --- | --- | --- |
| Clean Absorbents | Sanitary pads, cleaned cloth or reusable pads | What was the most commonly absorbent material used during the last 6 cycles? | 1 = Disposable sanitary pads 2 = Reusable cloths/towel 3 = Nothing 88 = Other | Not included |
|  | Wash with water and soap | With what do you wash your cloth? | 1 = Water only 2 = Water and soap/detergent 88 = Other | Not included |
|  | Dried outside | After washing it, how do you dry the cloth? | 1 = Dry it in the sun or open space 2 = Dry it inside the house 3 = I don't dry it 88 = Other | Not included |
|  | Stored with clothes | Where do you normally store the cloth for use next time? | 1 = With my clothes 2 = In some place in the toilet 3 = In some place of the changing room or hidden place inside house 4 = In hidden place outside house 88 = Other | Not included |
|  | Wrapped in polyethene or material | Do you wrap the cloth in anything when storing? | 1 = Yes, polythene 2 = Yes, other material 3 = No | Not included |
| Adequate frequency of absorbent change | Every 2 - 6 hours 24/6 = 4 x per day | How often do you change your absorbent material on your heaviest day? | 1,2,3,4,5,6,7 | 0 = 1 - 2  1 = 3 - 7 |
| Washing the body practices | Full body washing | What type of washing (bath or vaginal wash) do you practice during menstruation? | 1 = Only vaginal wash 2 = Bath of full body 3 = I don't wash myself | 0 = I don't wash myself  1 = Only vaginal washing & Bath of full body |
|  | Wash regularly throughout the cycle | How often do you wash yourself (bath or vaginal wash) during menstruation? | 1 = I don't wash myself 2 = Only the first day of my cycle 3 = A few times throughout the cycle 4 = At least once every day 5 = More than once everyday | 0 = I don't wash myself, only the first day of my cycle  1 = A few times throughout the cycle, at least once every day & more than once everyday |
|  | Wash the body with soap and water | What do you use to wash yourself during menstruation? | 1 = Water only 2 = Water and soap/detergent 88 = Other | Not included |
| Difficulty with disposal | Able to dispose of absorbent | How often have you experienced any of the following during your last two menstrual periods? Had difficulty finding a place to dispose of cloth or pad | 1 = Never 2 = Sometimes 3 = Often 4 = Always | Not included |
| Privacy for managing menstruation | Change absorbent in a private place | Where do you most often change your absorbent material when at home? | 1 = In household toilet 2 = Bathing room 3 = In toilet of neighbor/relative 4 = In private room in the house 5 = Outside (field/river/pond etc.) 88 = Other | 0 = Outside  (field/river/pond etc.) & Other  1 = In household toilet, bathing room, in toilet of neighbor/relative & in private room in the house |
|  | Wash absorbent in a private place | Where do you wash the absorbent materials you reuse? | 1 = Inside toilet stall 2 = Bathing room 3 = At private tube well/ tap in yard or house 4 = At public tube well/ tap in village 5 = In pond/ river 6 = I do not wash it/ Not applicable 88 = Other | 0 = At public tube well/ tap in village, in pond/ river, I do not wash it/ Not applicable & other  1 = Inside toilet stall, bathing room & at private tube well/ tap in yard or house |

## **Supplementary table 2:** Missing data.

|  | Control | Intervention | Total | P-value |
| --- | --- | --- | --- | --- |
| Variable | **Missing data (%)** | **Missing data (%)** | **Missing data (%)** |  |
| Age (Years) | 49 (9.28) | 39 (7.54) | 88 (8.42) | 0.61 |
| Wealth index | 122 (23.11) | 91 (17.60) | 213 (20.38) | 0.001 |
| Female caregiver education attainment | 81 (15.34) | 53 (10.25) | 134 (12.82) | <0.0001 |
| Experience of taboo | 5 (0.95) | 1 (0.19) | 6 (0.57) | 0.018 |
| Healthcare decisions | 76 (14.39) | 60(11.61) | 136 (13.01) | 0.47 |
| Market access | 76 (14.39) | 61 (11.80) | 137 (13.11) | 0.33 |
| Marital Status | 0 (0.00) | 0 (0.00) | 0 (0.00) | 0.30 |
| Religion | 72 (13.64) | 58 (11.22) | 130 (12.44) | 0.48 |
| WASH | 0 (0.00) | 0 (0.00) | 0 (0.00) | <0.0001 |
| MHM (Full definition) | 0 (0.00) | 0 (0.00) | 0 (0.00) | 0.020 |
| MHM (Relaxed definition) | 0 (0.00) | 0 (0.00) | 0 (0.00) | <0.0001 |
| The combined UGI variable | 0 (0.00) | 0 (0.00) | 0 (0.00) | 0.64 |
| MHM: Adequate frequency of absorbent change | 5 (0.95) | 1 (0.19) | 6 (0.57) | 0.15 |
| MHM: Type of washing | 5 (0.95) | 1 (0.19) | 6 (0.57) | 0.19 |
| MHM: Regularity of washing | 10 (1.89) | 3 (0.58) | 13 (1.24) | 0.16 |
| MHM: Privacy to change absorbent | 5 (0.95) | 1 (0.19) | 6 (0.57) | 0.0001 |
| MHM: Privacy to wash cloth | 70 (13.26) | 105 (20.31) | 175 (16.75) | <0.0001 |
| UGI: Abnormal discharge | 0 (0.00) | 0 (0.00) | 0 (0.00) | 0.92 |
| UGI: Burning or itching in genitalia | 0 (0.00) | 0 (0.00) | 0 (0.00) | 0.027 |
| UGI: Urinate frequently | 0 (0.00) | 0 (0.00) | 0 (0.00) | 0.51 |
| UGI: Burning or itching when urinating | 0 (0.00) | 0 (0.00) | 0 (0.00) | 0.81 |

#### **Supplementary table 3**. Unadjusted and adjusted estimates of selected characteristics and the ‘Adequate menstrual hygiene’ definition among women living in Odisha, India during July – October 2016 estimated by multilevel mixed-effects logistic regression (N=1045).

| Selected Characteristics | |  |  | Adequate MHM definition | | | | | |
| --- | --- | --- | --- | --- | --- | --- | --- | --- | --- |
|  |  |  | **Unadjusted^2^** | | | | **Adjusted^3^** | | |
|  |  | **n event/N^4^** | **% event** | **OR** | **95% CI** | **P-value^5^** | **OR^7^** | **95% CI** | **P-value^5^** |
| Age (years)  *(n=957)* | 18 – 24 | 32/288 | 11.11 | 1 |  | 0.22 | 1 |  | 0.18 |
|  | 25 – 29 | 45/385 | 11.69 | 1.09 | 0.66-1.82 |  | 1.56 | 0.86 – 2.82 |  |
|  | 30 + | 20/284 | 7.04 | 0.67 | 0.36-1.23 |  | 0.91 | 0.43 – 1.94 |  |
| Wealth Index  *(n=832)* | Poor/Middle | 51/623 | 8.19 | 1.00 |  | <0.001 | 1 |  | 0.03 |
|  | Rich | 38/209 | 18.18 | 2.52 | 1.55-4.09 |  | 1.88 | 1.08 – 3.26 |  |
| Caregiver Education Attainment  *(n=911)* | Primary or less | 20/368 | 5.43 | 1 |  | <0.001 | 1 |  | 0.01 |
|  | Secondary or above | 72/543 | 13.26 | 2.71 | 1.58—4.64 |  | 2.33 | 1.20 – 4.53 |  |
| Experience Stigma^6^  *(n=1,039)* | No | 62/689 | 9.00 | 1 |  | 0.16 | 1 |  | 0.07 |
|  | Yes | 38/350 | 10.86 | 1.38 | 0.88-2.18 |  | 1.65 | 0.96 – 2.85 |  |
| Market Access^7^  *(n=908)* | No | 27/230 | 11.74 | 1 |  | 0.5 | 1 |  |  |
|  | Yes | 66/678 | 9.73 | 0.84 | 0.51-1.38 |  | 0.79 | 0.45 – 1.37 | 0.40 |
| Healthcare Decision^8^  *(n=909)* | Self | 19/257 | 7.39 | 1 |  | 0.05 | 1 |  |  |
|  | Someone else | 41/306 | 13.40 | 2.05 | 1.12-3.73 |  | 1.97 | 0.98 – 3.94 |  |
|  | Self and someone else (joint) | 33/346 | 9.54 | 1.14 | 0.76-2.59 |  | 1.30 | 0.63 – 2.67 | 0.12 |
| ^1^MHM Relaxed definition: Adequate frequency of absorbent change, wash body with soap and water (frequency and type of washing only) and privacy for managing menstruation.  ^2^Adjusted for clustering at the pair and village level  ^3^Adjusted for clustering at the pair and village level and for other selected characteristics in the table (age, wealth index, caregiver education attainment and healthcare decision)  ^4^ Numbers do not add up to 100% due to missing values. Not all women responded to the HH survey as the HH survey targeted the mother or primary caregiver of the youngest child <5.  ^5^P-values derived from nested likelihood ratio tests  ^6^Menstruating women who experienced stigma from others during the last two menstruation cycles.  ^7^The number of times menstruating women have attended the market. This indicates access to resources such as absorbents.  ^8^Independence on healthcare decision making. This indicates ease of access to healthcare for women. | | | | | | | | | |

**Supplementary Table 4.** Self-reported symptoms of diseases pertaining to UGS and the relationship with WASH intervention by menstruating females in India, Odisha during July – October 2016 (n=1,045).

| Survey question | Survey responses | Women living in control villages  *(n=528)* | | Women living in intervention villages  *(n=517)* | |
| --- | --- | --- | --- | --- | --- |
|  |  | **n** | **%** | **n** | **%** |
| In the last two weeks, did you have abnormal vaginal discharge? | No | 478 | 90.53 | 469 | 90.72 |
|  | Yes | 50 | 9.47 | 48 | 9.28 |
| In the last two weeks, do you have a feeling of burning or itching in the genitalia (vulvovaginal area)? | No | 498 | 94.32 | 502 | 97.10 |
|  | Yes | 30 | 5.68 | 15 | 2.90 |
| In the last two weeks, do you feel the need to urinate more frequently? | No | 501 | 94.89 | 495 | 95.74 |
|  | Yes | 27 | 5.11 | 22 | 4.26 |
| In the last two weeks, do you have a feeling of burning or itching or pain when urinating? | No | 506 | 95.83 | 497 | 96.13 |
|  | Yes | 22 | 4.17 | 20 | 3.87 |
| ^1^ χ^2^ test |  |  |  |  |  |

#### **Supplementary table 5.** Unadjusted and adjusted estimates of selected characteristics and UGS among women living in Odisha, India during July – October 2016 estimated by multilevel mixed-effects logistic regression (N=1045).

| Selected characteristics | |  | UGS variable^1^ | | | | | |
| --- | --- | --- | --- | --- | --- | --- | --- | --- |
|  |  |  | **Unadjusted**^2^ | | | **Adjusted**^3^ *(n=711)* | | |
|  |  | **n event/N^4^** | **OR** | **95% CI** | **P-value**^5^ | **OR** | **95% CI** | **P-value**^5^ |
| Age (years)  *(n=957)* | 18 – 24 | 46/288 | 1 |  | 0.4 | 1 |  | 0.49 |
|  | 25 – 29 | 50/385 | 0.79 | 0.51 – 1.24 |  | 0.76 | 0.45 – 1.28 |  |
|  | 30 + | 48/284 | 1.07 | 0.67 – 1.69 |  | 0.98 | 0.56 – 1.72 |  |
| Wealth Index  *(n=832)* | Poor/Middle | 91/623 | 1 |  | 0.8 | 1 |  | 0.77 |
|  | Rich | 26/209 | 0.93 | 0.57 – 1.53 |  | 0.92 | 0.53 – 1.59 |  |
| Caregiver Education Attainment  *(n=911)* | Primary or less | 53/368 | 1 |  | 0.8 | 1 |  | 0.42 |
|  | Secondary or above | 82/543 | 1.05 | 0.71 – 1.56 |  | 1.22 | 0.75 – 1.98 |  |
| Experience Taboo^6^  *(n=1,039)* | No | 92/689 | 1 |  | 0.08 | 1 |  | 0.05 |
|  | Yes | 63/350 | 1.39 | 0.97 – 1.99 |  | 1.56 | 1.00 – 2.43 |  |
| Market Access^7^  *(n=908)* | No | 41/230 | 1 |  | 0.2 | 1 |  | 0.38 |
|  | Yes | 94/678 | 0.76 | 0.50 – 1.15 |  | 0.80 | 0.49 – 1.30 |  |
| Healthcare Decision^8^  *(n=909)* | Self | 47/257 | 1 |  | 0.1 | 1 |  | 0.17 |
|  | Someone else | 38/306 | 0.61 | 0.38 – 0.99 |  | 0.59 | 0.34 – 1.04 |  |
|  | Self and someone else (joint) | 50/346 | 0.73 | 0.47 – 1.16 |  | 0.69 | 0.40 – 1.17 |  |
| ^1^ The UGS variable consists of self-reported symptoms in the past two weeks of abnormal vaginal discharge, burning or itching in the genitalia, burning or itching when urinating and urinating frequently.  ^2^Adjusted for clustering at the pair and village level  ^3^Adjusted for clustering at the pair and village level and for other selected characteristics in the table.  ^4^ Numbers do not add up to 100% due to missing values. Data was missing because not all women responded to the HH survey as the HH survey targeted the mother or primary caregiver of the youngest child<5. Missing data were explored to investigate patterns and difference between the intervention groups using Chi square (Supplementary Table 2).  ^5^ P-values derived from nested likelihood ratio tests  ^6^Menstruating women who experienced taboo from others during the last two menstruation cycles.  ^7^The number of times menstruating women have attended the market. This indicates access to resources such as absorbents.  ^8^Independence on healthcare decision making. This indicates ease of access to healthcare for women. | | | | | | | | |
